# The radiosensitizing effect of β-Thujaplicin, a tropolone derivative inducing S-phase cell cycle arrest, in head and neck squamous cell carcinoma-derived cell lines

**DOI:** 10.1007/s10637-022-01229-3

**Published:** 2022-04-12

**Authors:** Markus Haas, Teresa Lenz, Lorenz Kadletz-Wanke, Gregor Heiduschka, Bernhard J Jank

**Affiliations:** Department of Otorhinolaryngology, Medical University of Vienna, Währinger Gürtel 18-20, 1090 Vienna, Austria

**Keywords:** β-Thujaplicin, Head and neck squamous cell carcinoma, cancer, HNSCC, Radiosensitization

## Abstract

**Background:**

Resistance to radiotherapy is a common cause of treatment failure in advanced head and neck squamous cell carcinoma (HNSCC). ß-Thujaplicin, a natural tropolone derivative, acts as an anti-cancer agent and has recently been shown to radiosensitize non-HNSCC cancer cells. However, no data is currently available on its radiosensitizing potential in HNSCC.

**Methods:**

To investigate the effect of ß-Thujaplicin and irradiation in HNSCC cell lines CAL27 and FADU, we performed a cell viability assay, colony forming assay, flow cytometry for cell cycle analysis and a wound healing assay. Drug-irradiation interaction was analyzed using a zero-interaction potency model.

**Results:**

Treatment with ß-Thujaplicin led to a dose-dependent decrease in cell viability and enhanced the effect of irradiation. Clonogenic survival was inhibited with synergistic drug-irradiation interaction. ß-Thujaplicin further led to S-phase arrest and increased the sub-G1 population. Moreover, combined ß-Thujaplicin and irradiation treatment had a higher anti-migratory effect compared to irradiation alone.

**Conclusions:**

ß-Thujaplicin acts as a radiosensitizer in HNSCC cell lines. Further evaluation of its use in HNSCC therapy is warranted.

## Introduction

Worldwide, head and neck squamous cell carcinoma (HNSCC) is the seventh most common malignancy and in 2018 over 890,000 new cases and 450,000 deaths have been reported [1]. Common risk factors for developing HNSCC include tobacco consumption and alcohol abuse as well as Human papillomavirus (HPV) infection [2]. In western nations, the incidence of HPV-positive disease has increased considerably in recent decades, while the number of HPV-negative HNSCC is declining, possibly as a result from the decreasing prevalence of tobacco consumption [3, 4]. Although survival rates of HPV-positive HNSCC have been steadily increasing since the late 1990s leading to efforts for treatment de-escalation, the prognosis of HPV-negative disease lacks similar improvements [4]. Importantly, resistance to radiotherapy is a key contributor to treatment failure and occurs more commonly in HPV-negative disease [5]. A combination of radiotherapy and chemotherapy is a standard treatment regimen in advanced HNSCC. Platinum-based agents such as cisplatin are effective in improving response to irradiation [6]. However, the toxicity associated with platinum-based agents is severe and can lead to failure to complete treatment. Moreover, not all HNSCC cases are sensitive to platin-based chemoradiotherapy [7]. Hence, radiosensitizers with a better toxicity profile and a more consistent treatment response are urgently needed.

ß-Thujaplicin, also known as Hinokitiol, is a tropolone derivative and natural product isolated from the heartwood of cupressaceous trees [8]. Due to its antimicrobial properties, ß-Thujaplicin is used in oral hygiene products, such as dentifrice, and as food additive to extend shelf life of fresh products [9–11]. ß-Thujaplicin has further been shown to have a growth inhibitory effect on various cancer cell lines [12–14]. ß-Thujaplicin exerts its anticancer activity through a wider variety of mechanisms, including induction of cell cycle arrest & programmed cell death [14–16], inhibiting cancer cell migration and metastasis [17, 18], impairing DNA damage response by inhibiting homologous recombination [19], epigenetic modification by inhibition of DNA methyltransferase 1 [20] and targeting of cancer stem cells by RNA interference [21] and inhibition of vasculogenic mimicry [22]. Molecular mechanisms of radioresistance involve dysregulation of such pathways related to cell cycle control [23], DNA damage response [24] and DNA methylation [25], which are targeted by ß-Thujaplicin, making it a promising candidate for improving radiosensitivity in HNSCC. In osteosarcoma cell lines, the radiosensitizing effect of ß-Thujaplicin has recently been demonstrated [19].

Regarding HNSCC, β-Thujaplicin has been investigated in a drug screening study of hydroxyketone chelators and was shown to exhibit potent cytotoxic activity in oral squamous cell carcinoma and submandibular gland carcinoma cell lines with high tumor specificity compared to patient-derived normal oral fibroblast and pulp cells [26]. Furthermore, in a HNSCC xenograft model, β-Thujaplicin led to a significant reduction in tumor weight after one month of treatment [27].

However, no data is available regarding combined use of ß-Thujaplicin and irradiation in HNSCC. In this study, we aimed to investigate the potential radiosensitizing effect of ß-Thujaplicin in two HNSCC cell lines and its effects on cell viability, clonogenic survival, cell cycle and migration.

## Methods

### Cell culture and reagents

To investigate the effect of β-Thujaplicin in HNSCC, two HPV-negative HNSCC cell lines, namely CAL27 and FADU, were obtained from the American Type Culture Collection (ATCC, Manassas, Virginia, USA). Adherent cell cultures were maintained in 10 cm dishes and incubated at 37 °C and 5% CO_2_ using Dulbecco’s Modified Eagle Medium (DMEM) supplemented with 10% of fetal bovine serum (FBS) and 1% penicillin-streptomycin (Thermo Fischer Scientific, Waltham, Massachusetts, USA). For passaging, 0.05% 1X Trypsin/EDTA (Thermo Fischer Scientific, Waltham, Massachusetts, USA) was used for cell detachment and cells were centrifuged at 340 g for 5 min. Cells were discarded once passage number 30 was reached. β-Thujaplicin was purchased from Selleck Chemicals (Houston, Texas, US) in solid form, dissolved in dimethylsulfoxid (DMSO) and stored at -20 °C until use. DMSO was used as a vehicle control at a concentration of 0.1%. Dilutions of the β-Thujaplicin stock solution were kept at final DMSO concentrations below 0.1%.

### Radiation treatment

Cell lines were exposed to X-ray radiation generated by a YXLON Maxishot unit (Yxlon International X-ray GmbH, Hamburg, Germany) at a fixed focus object distance of 45.5 cm with a tube voltage of 200 kV and current of 20 mA. The focus size was 5.5 mm and a filter composed of 4 mm aluminum and 0.6 mm copper was used. Cells were irradiated with doses ranging from 2 to 8 Gy at 1 Gy/min.

### Cell viability assay

A resazurin-based colorimetric assay was used to determine cell viability. Resazurin sodium salt was obtained from Sigma-Aldrich (St. Louis, Missouri, USA) and a 10X stock solution was prepared at a concentration of 5.6 mM with phosphate-buffered saline as a solvent. A final concentration of 560 µM was used for experiments, which has previously been determined to be a concentration similar to commercial agents [28].

Cells were seeded at a density of 6 × 10^3^ cells/well in 96 well plates with six replicates per inhibitor concentration. Cells were treated 24 h after seeding for a duration of 72 h. Irradiation was performed immediately after β-Thujaplicin was added. Culture medium with β-Thujaplicin was removed 72 h after exposure and a 9:1 mixture of culture medium and resazurin solution was added. After cells were incubated for 1 h, absorbance detection was performed using a Tecan Spark microplate reader (Tecan Group Ltd., Maennedorf, Switzerland) at wavelengths of 570 and 600 nm. Results were normalized to an untreated vehicle control group and reported as percentage of cells viable. The experiment was repeated three times.

### Migration assay

In order to determine the effects of β-Thujaplicin on cell migration in HNSCC, a modified version of the wound healing assay was used. Instead of creating a gap by inflicting a wound with a pipette tip to a confluent monolayer, 2-well silicone culture-inserts (ibidi GmbH, Graefelfing, Germany) were used to create two separate cell layers with a uniform gap measuring 500 μm in width.

24 well plates were prepared by placing the adhesive side of the culture-inserts into each well, exposing the plate to UV-light in a laminar flow unit for 1 h and storing the plate in the incubator overnight. On the next day, cells were seeded into both wells of the inserts at densities of 3 × 10^4^ and 1.5 × 10^4^ cells/well for CAL27 and FADU, respectively. Each treatment group consisted of six replicates.

24 h after seeding, cells were irradiated with 4 Gy. Next, cells were incubated for another 24 h before β-Thujaplicin was added. Cell migration was initiated 48 h post-irradiation by removing the inserts. Images were taken using a Tecan Spark microplate reader with life cell imaging function at 0 and 20 h after insert removal. β-Thujaplicin treatment was continued during cell migration resulting in a total incubation time of 44 h. Images were analyzed using ImageJ software (U.S. National Institutes of Health, Bethesda, Maryland, USA) and the MRI Wound Healing Tool plugin (https://github.com/MontpellierRessourcesImagerie/imagej_macros_and_scripts/wiki/Wound-Healing-Tool) to detect the cell-free gap area. Results were reported as percentage of the reduction in gap area after 20 h. The experiment was repeated four times.

### Colony forming assay

Cells were seeded in 12 well plates at low densities to allow for colony formation with minimal overlap of individual colonies. Seeding densities were increased with treatment escalation in line with an established protocol [29]. Cells were seeded ranging from 2 × 10^2^ to 1.6 × 10^3^ cells/well with three replicates per treatment group. After 24 h, cells were treated with β-Thujaplicin and irradiated. 72 h post-exposure, the inhibitor-containing medium was replaced by drug-free culture medium and cells were incubated for an additional 10 days.

Life cell imaging using a Tecan Spark microplate reader was utilized for measurement to create images of every well. The images were then analyzed using ImageJ by enhancing contrast and creating a binary image. The “Analyze particles” function of ImageJ was used to count the number of colonies per well. A cut-off value for minimum particle size was determined by measuring the average pixel area of colonies that were visually confirmed to consist of 50–60 cells. As a result, only colonies that consisted of at least 50 cells or more were counted.

Results are reported as surviving fraction and normalized to the control group. To determine the surviving fraction, the number of colonies formed was multiplied by number of cells seeded and divided by plating efficiency (i.e. the percentage of cells seeded per well that formed colonies in the vehicle group). The experiment was repeated three times.

### Flow cytometry

Flow cytometry was employed to determine the effects of β-Thujaplicin on the cell cycle. Flow cytometry analysis was performed according to an established protocol using 4′,6-Diamidin-2-phenylindol (DAPI) as a DNA stain to quantify every cell’s DNA content and assign cell cycle phases (G1, S, G2) [30].

Cells were seeded in 6 well plates as duplicates at densities of 3 × 10^5^ and 1.5 × 10^5^ cells/well for CAL27 and FADU, respectively. After 24 h, cells were treated with a β-Thujaplicin dose of 5 µM and 10 µM, irradiated at 4 Gy and then incubated for an additional 72 h. After treatment, cells were fixated with 70% ethanol for 30 min at 4 °C and then stained with a solution of DAPI and Triton-X at a ratio of 1:1,000 for 10 min at room temperature.

Cells were analyzed using a BD LSRFortessa cytometer (BD, Franklin Lakes, New Jersey, USA). A violet laser (405 nm) was used for excitation of DAPI and a 450/50 bandpass filter for detection. During gating, doublet discrimination was performed. FlowJo software (FlowJo LLC, Ashland, Oregon, USA) was used to assign cells to G1-, S- and G2-phase according to the Watson pragmatic algorithm. Cells with DNA content below that of the G1 population were identified as sub-G1 population. The sub-G1 population contains cells with fractional DNA content resulting from DNA fragmentation during apoptosis and accumulation of apoptotic bodies [31]. The experiment was repeated three times.

### Statistical analysis

Statistical analysis for cell viability, migration and cell cycle was performed using two-way analysis of variance (ANOVA) for the two factors inhibitor and irradiation. ANOVA was followed up with post-hoc testing using Bonferroni’s correction for multiple comparisons. Half maximal inhibitory concentrations (IC_50_) were calculated using a non-linear regression curve fit. All tests were two-tailed and the significance level was set at 5%. GraphPad Prism 8 software (GraphPad Software, Inc., San Diego, California, USA) was used for calculations and visual representations of the data. For cell viability and clonogenic survival, the Synergyfinder 2.0 web-app (https://synergyfinder.fimm.fi) by Ianevski et al. was utilized to investigate the drug-irradiation interaction and calculate synergy scores according to the zero-interaction potency (ZIP) model [32]. ZIP scores > 10 are interpreted as synergistic, scores between 10 and − 10 as additive and scores below − 10 as antagonistic.

## Results

### β-Thujaplicin reduces cell viability in a dose dependent manner with additive drug-irradiation interaction

**Fig. 1:**
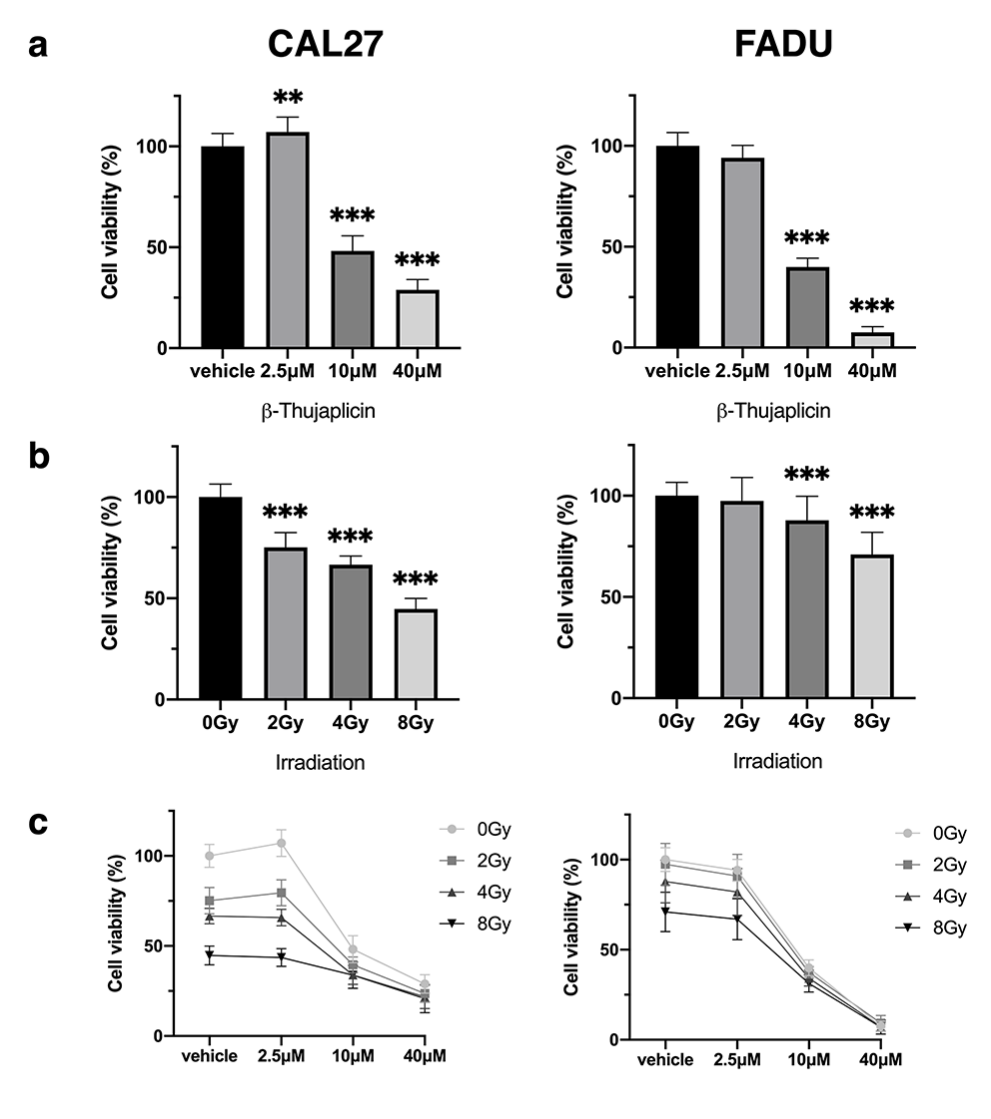
**β-Thujaplicin decreases cell viability in a dose dependent manner.** CAL27 and FADU were irradiated with 0 Gy, 2 Gy, 4 Gy & 8 Gy and treated with 0.1% DMSO (vehicle) and 2.5 µM, 10 µM & 40 µM β-Thujaplicin for 72 h. Cell viability was determined with a resazurin-based colorimetric assay. Data is expressed as percentage of cell viability normalized to the control group (vehicle, 0 Gy). Cell viability in response to increasing doses of **(a)** β-Thujaplicin, **(b)** irradiation and **(c)** combination treatment is shown. Post-hoc testing was performed to compare treatment groups to the control group. Bar graphs report mean values ± SD and significance levels (* p < 0.05, ** p < 0.01, *** p < 0.001). The experiment was repeated three times with six replicates per condition

To assess cell viability, HNSCC cell lines CAL27 and FADU were treated with increasing doses of β-Thujaplicin, ranging from 2.5 µM to 40 µM, and a resazurin-based colorimetric assay was performed. β-Thujaplicin reduced cell viability in a dose-dependent manner (Fig. 1a). IC_50_-values were calculated at 15.1 µM (12.6–18.3 µM, CI 95%) and 8.5 µM (7.4–9.8 µM, CI 95%) for CAL27 and FADU, respectively.

Next, we aimed to determine baseline radiosensitivity for each cell line. We, therefore, treated cells with irradiation doses ranging from 2 to 8 Gy (Fig. 1b). At the lowest radiation dose, CAL27 already exhibited a 24.8% (19.9–29.7%, CI 95%) decrease in cell viability, while FADU only showed a non-significant decrease of 2.6% (-3.9-9.1%, CI 95%). At higher radiation doses of 4 and 8 Gy, both cell lines exhibited significant reductions in cell viability.

To evaluate a radiosensitizing effect of ß-Thujaplicin, cells were treated with β-Thujaplicin in combination with irradiation (Fig. 1c). At doses of 10 µM and 40 µM, cell viability was uniformly decreased across all radiation doses when compared to vehicle control of the respective radiation dose. Drug-irradiation interaction was assessed using a ZIP model to determine additive or synergistic interaction between β-Thujaplicin and irradiation. Combination treatment had an additive effect on the reduction of cell viability with cumulative ZIP synergy scores of 1.6 (± 4.7, SD) and 2.9 (± 6.2, SD) for CAL27 and FADU, respectively.

### β-Thujaplicin decreases clonogenic survival in a dose-dependent manner with synergistic drug-irradiation interaction

**Fig. 2:**
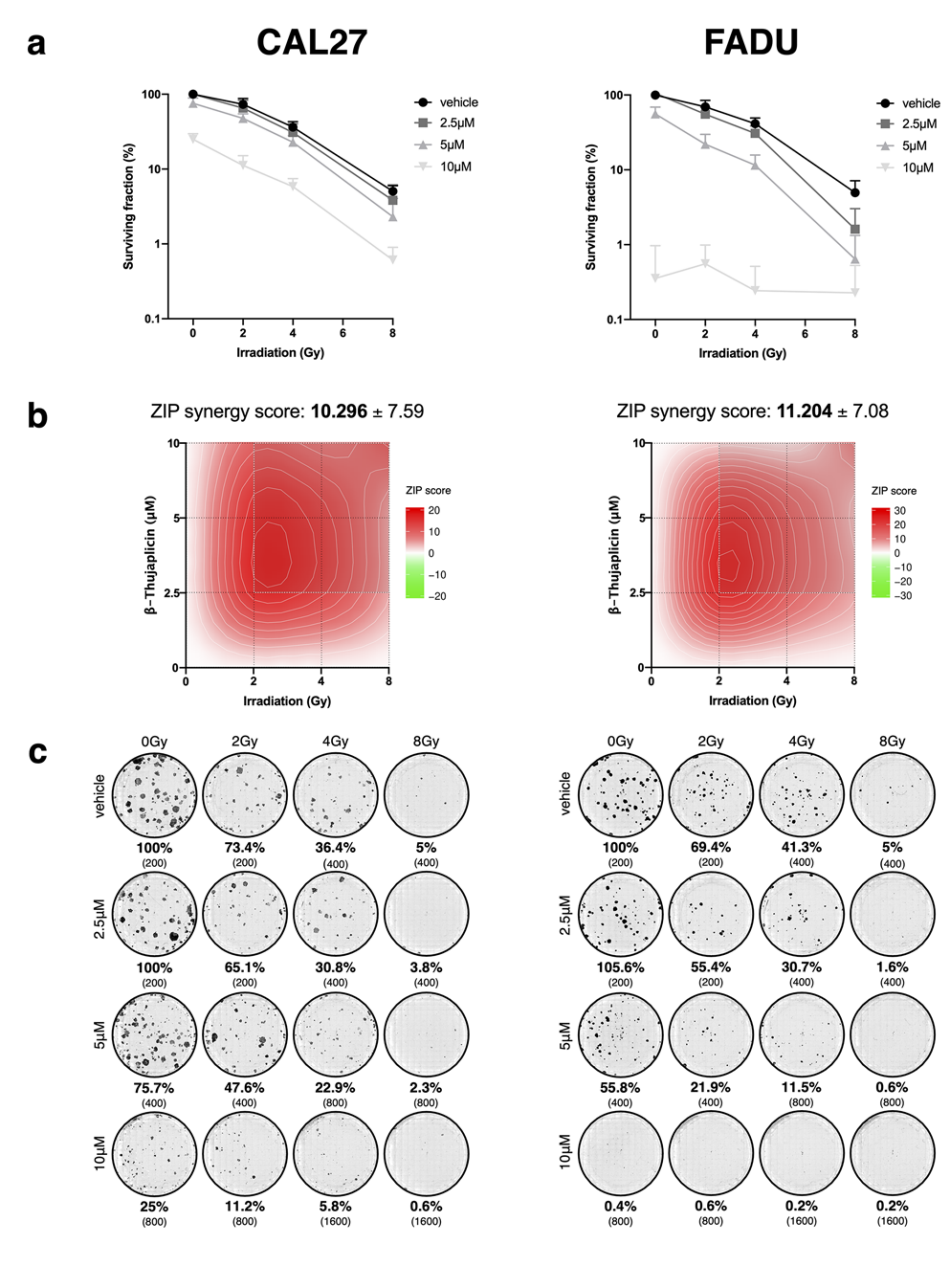
**β-Thujaplicin inhibits colony formation in a dose-dependent manner.** CAL27 and FADU were irradiated with 0 Gy, 2 Gy, 4 Gy & 8 Gy and treated with 0.1% DMSO (vehicle) and 2.5 µM, 5 µM & 10 µM β-Thujaplicin for 72 h. β-Thujaplicin was then replaced by culture medium and cells were allowed to incubate for an additional 10d. The number of cells seeded was escalated according to increasing treatment intensity and the surviving fraction was normalized to the control group (vehicle, 0 Gy). The experiment was repeated three times with three replicates per condition. **(a)** Point graphs report mean values ± SD. **(b)** Synergy maps are shown and ZIP synergy summary scores ± SD are reported, which are defined as the average excess response caused by drug/radiation interactions based on individual ZIP scores for all dose combinations. Scores above 10 indicate synergistic interaction, between 10 and − 10 additive interaction and below 10 antagonistic interaction. **(c)** Images of cell colonies are shown. The surviving fraction is reported as mean values and the number of cells seeded per condition are shown in parentheses

In order to assess the effect of β-Thujaplicin on clonogenic survival and its potential additive or synergistic effect with irradiation, a colony formation assay was performed at doses ranging from 2.5 µM to 10 µM and radiation doses from 2 to 8 Gy (Fig. 2a).

β-Thujaplicin exhibited a dose-dependent inhibitory effect on clonogenic survival in CAL27 and FADU. At 10 µM, the surviving fraction was reduced to 25.0% (± 4.7%, SD) and to below 1% (± 0.6%, SD) in non-irradiated CAL27 and FADU, respectively. With combination treatment, the cumulative ZIP synergy scores were 10.3 (± 7.6, SD) for CAL27 and 11.2 (± 7.1, SD) for FADU, indicating a synergistic, radiosensitizing effect of β-Thujaplicin on clonogenic survival (Fig. 2b). Analysis of individual ZIP scores for every dose combination revealed that the synergistic effect was most pronounced at dose ranges of 2 to 4 Gy and 2.5 µM to 5 µM for irradiation and β-Thujaplicin, respectively. (Table 1)

**Table 1:** Individual ZIP synergy scores for every dose combination

β-T (µM)	IRR (Gy)	CAL27		FADU	
		**ZIP score** ^**a**^	**SD**	**ZIP score** ^**a**^	**SD**
2.5	2	**19.07**	± 18.04	**29.35**	± 13.55
	4	**13.50**	± 9.58	**13.99**	± 7.85
	8	2.97	± 2.32	5.95	± 1.43
5	2	**17.39**	± 7.07	**24.48**	± 7.83
	4	**12.72**	± 5.01	**13.52**	± 4.22
	8	2.77	± 1.75	3.05	± 0.69
10	2	8.98	± 3.92	-0.41	± 0.43
	4	4.98	± 1.63	-0.29	± 0.27
	8	0.86	± 0.29	-0.28	± 0.30

Abbreviations: β-T, β-Thujaplicin; IRR, irradiation; ZIP, zero interaction potency

^a^ ZIP scores above 10 indicate synergistic interaction (highlighted in red), between 10 and − 10 additive interaction and below − 10 antagonistic interaction

### β-Thujaplicin leads to S-phase cell cycle arrest and increases the sub-G1 population

To investigate the effect of β-Thujaplicin on cell cycle and apoptosis in HNSCC cell lines, cells were stained with a DNA stain (DAPI) and analyzed using flow cytometry and assigned to the cell cycle phases G1, S and G2 according to DNA content. Additionally, the sub-G1 population was quantified.

**Fig. 3:**
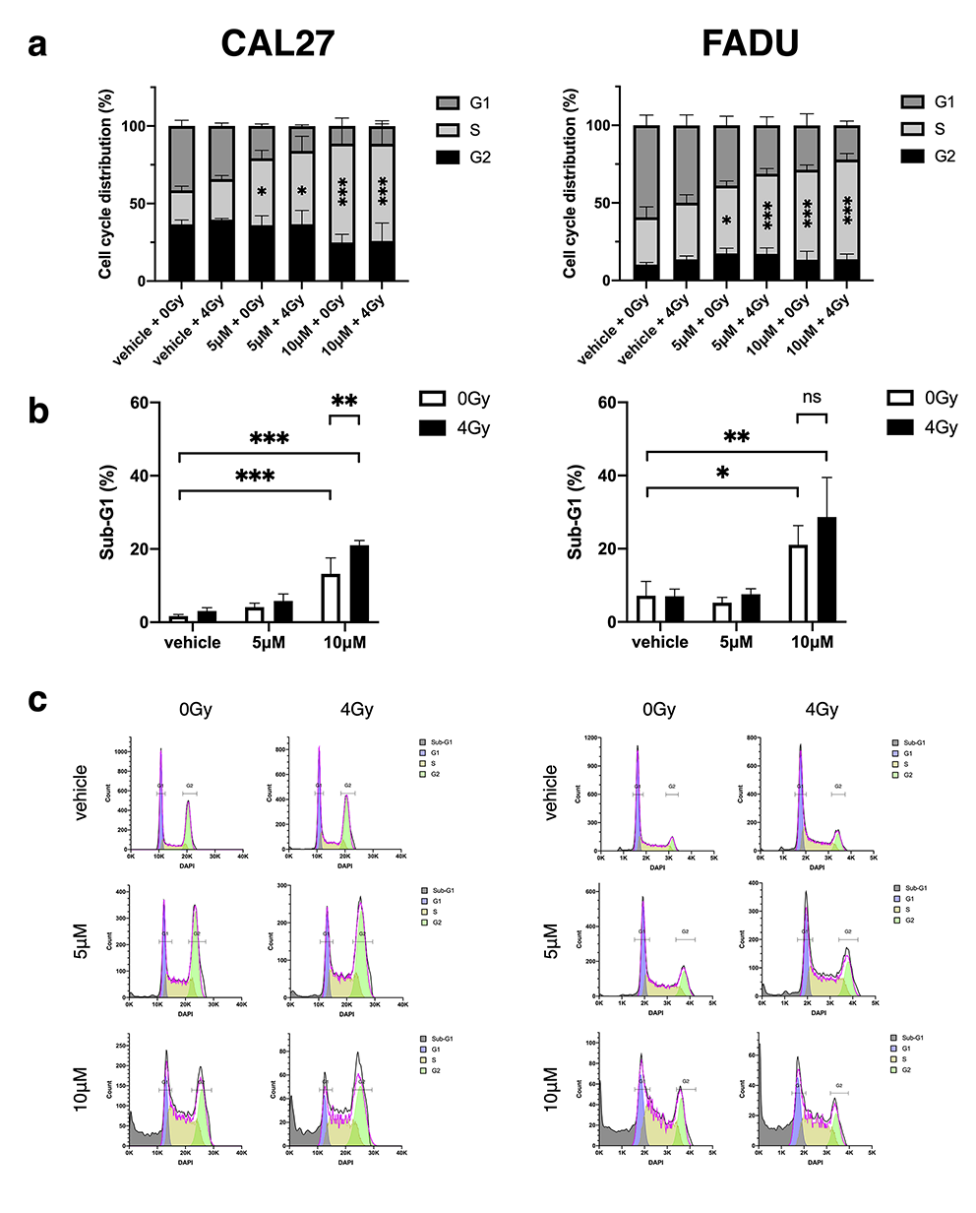
**β-Thujaplicin leads to S-phase arrest and increases the sub-G1 population.** CAL27 and FADU were irradiated with 0 Gy & 4 Gy and treated with 0.1% DMSO (vehicle) and 5 µM & 10 µM β-Thujaplicin for 72 h. Cells were fixated with 70% ethanol, stained with DAPI and analyzed using a flow cytometer. The experiment was repeated three times with two replicates per condition. **(a)** Cell cycle distribution is reported as percentage of the cell population in stages G1, S and G2. Significance levels are shown in comparison to the control group (vehicle, 0 Gy). **(b)** The sub-G1 population is reported as percentage of as cells with less DNA content compared to cells in G1, S and G2 phase. Bar graphs report mean values ± SD and significance levels (ns p > 0.05, * p < 0.05, ** p < 0.01, *** p < 0.001). **(c)** DNA content frequency histograms of all treatment groups are shown (grey = sub-G1, blue = G1, yellow = S, green = G2)

β-Thujaplicin led to S-phase cell cycle arrest in both cell lines at 5 µM and 10 µM (Fig. 3a). At 10 µM, the percentage of cells in S-phase was increased by 41.7% (21.7–62.7%, CI 95%) and 27.6% (16.8–38.5%, CI 95%) in CAL27 and FADU, respectively. With a dose-dependent increase in the S-phase population, the percentage of cells in G1 phase decreased proportionally. The G2 population was unaffected by β-Thujaplicin treatment. Irradiation with 4 Gy did not lead to significant changes in cell cycle distribution compared to the control group and combination treatment was not significantly different from β-Thujaplicin treatment alone.

The sub-G1 population was increased in both cell lines at β-Thujaplicin doses of 10 µM, but not at 5 µM (Fig. 3b). Irradiation with 4 Gy did not exhibit a significant effect compared to the control group. However, the sub-G1 population of CAL27 treated with both irradiation and 10 µM of β-Thujaplicin was increased by 7.7% (3.0-12.5%, CI 95%) compared to β-Thujaplicin alone. Similarly, combination treatment at 10 µM also led to an increase of 7.6% (-4.4-19.6%, CI 95%) in the sub-G1 population in FADU when compared to singular β-Thujaplicin treatment, but the result was not significant.

### β-Thujaplicin inhibits cell migration

**Fig. 4:**
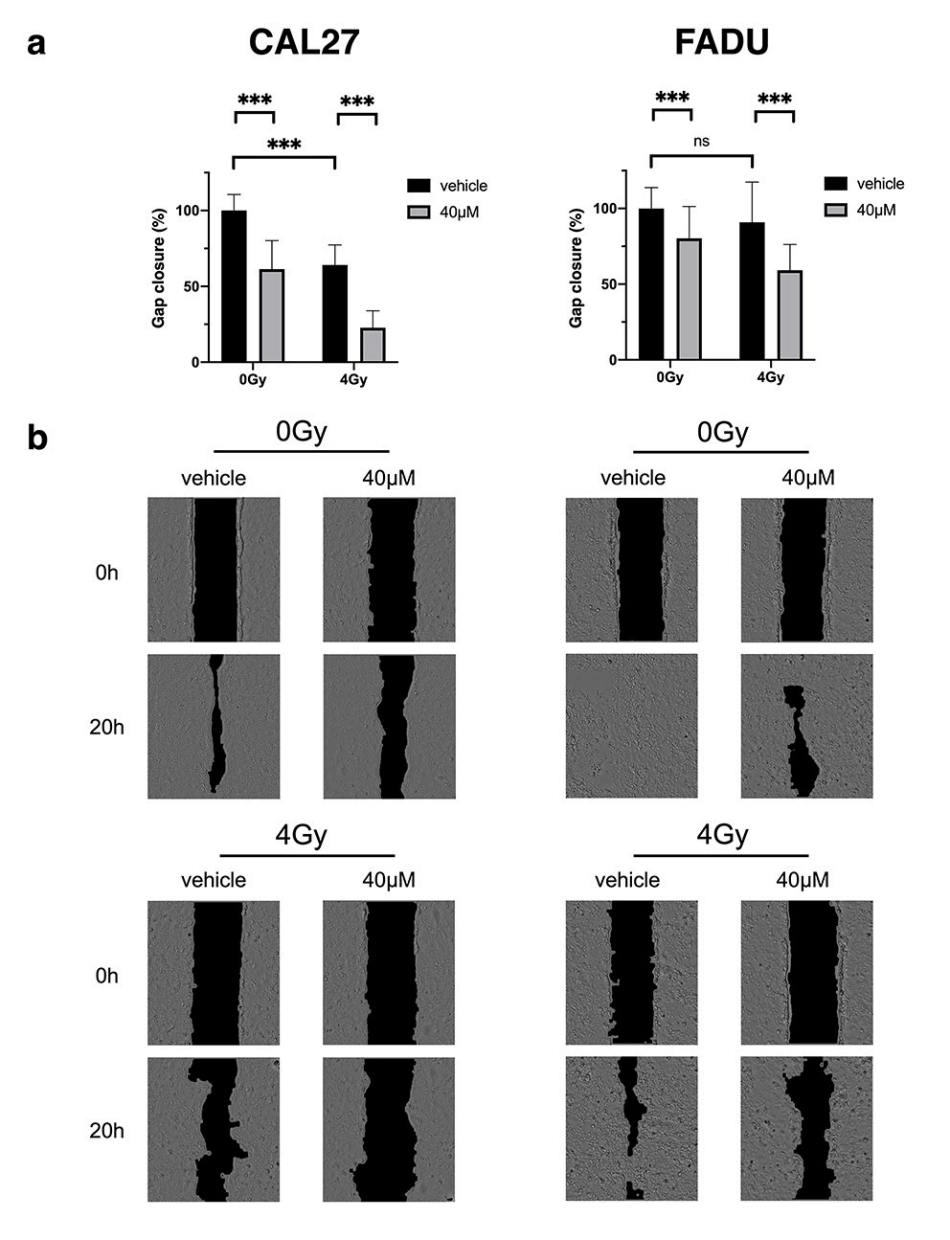
**β-Thujaplicin inhibits cell migration.** CAL27 and FADU were treated with 0.1% DMSO (vehicle) or 40 µM β-Thujaplicin for 44 h and irradiated with 0 or 4 Gy. Images were taken at 0 and 20 h after cell migration was initiated by removing culture-inserts. The experiment was repeated four times with six replicates per condition. **(a)** Percentage of gap closed indicates reduction in gap area normalized to the control group and is reported for all treatment groups. Bar graphs report mean values ± SD and significance levels (ns p > 0.05, * p < 0.05, ** p < 0.01, *** p < 0.001). **(b)** Images at 0 and 20 h after insert removal are shown

To determine the combined effect of β-Thujaplicin and irradiation on the migratory capability of HNSCC cell lines, an adapted wound healing assay using physical exclusion was performed. (Fig. 4) β-Thujaplicin inhibited cell migration in both cell lines. Irradiation at 4 Gy led to a decrease in cell migration by 35.9% (25.1–46.6, CI 95%) in CAL27, while FADU did not show a significant reduction in migration. With β-Thujaplicin and irradiation combined, inhibition of migration was significantly higher compared to single treatment with either treatment modality. Combination treatment reduced migration by an additional 41.4% (30.6–52.1%, CI 95%) in CAL27 and 31.7% (16.0-47.4, CI 95%) in FADU compared to irradiation alone.

## Discussion

Resistance to radiotherapy is a common cause of treatment failure in head and neck cancer therapy [5]. Therefore, great efforts have been made to enhance radioresponse by pharmacological means using radiosensitizers. Phytochemicals are an active field of investigation in cancer research, since plant-derived agents such as the antimitotic drugs docetaxel or etoposide have played an important role in cancer therapy for decades [33]. Recently, the natural tropolone derivative β-Thujaplicin has emerged as a novel candidate for cancer therapy with a favorable toxicity profile [34, 35]. In this study, we demonstrate that β-Thujaplicin enhances response to irradiation in HNSCC cell lines CAL27 and FADU.

ß-Thujaplicin exhibited a potent cytotoxic effect on HNSCC cells with IC_50_ concentrations below 20 µM. ß-Thujaplicin was previously shown to reduce cell viability in other cancer cell lines, such as colon cancer and melanoma, at similar doses. Interestingly, non-cancerous colon cells and melanocytes required considerably higher doses in order to affect cell viability [13, 20]. It has further been demonstrated that oral keratinocytes are less sensitive to ß-Thujaplicin compared to oral squamous cell cancer cells, suggesting a cancer-selective effect [36]. Importantly, as food additive, the acceptable daily intake (ADI) of β-Thujaplicin is 125ppm, which corresponds to a molar concentration of 762 µM [27]. Based on in silico analysis, β-Thujaplicin is predicted to have a high oral bioavailability [37]. Therefore, it can be reasonably assumed that the inhibitory effect of β-Thujaplicin on HNSCC cells occurs at clinically relevant concentrations that are considerably lower than the ADI. However, pharmacodynamic studies are needed to better understand the metabolization of β-Thujaplicin in vivo.

Regarding combination treatment, we found that β-Thujaplicin and irradiation led to a higher decrease in cell viability compared to irradiation alone with an additive drug-irradiation interaction. In terms of clonogenic survival, β-Thujaplicin led to considerable inhibition on colony formation at low doses and drug-irradiation interaction was synergistic. The longer incubation time of colony formation assays may have contributed to the enhanced radiosensitizing effect of β-Thujaplicin on clonogenicity compared to cell viability. Across all dose combinations, the synergistic effect was most pronounced at 2.5 µM and irradiation with 2 Gy. Interestingly, at this concentration, β-Thujaplicin alone had no inhibitory effect on either cell viability or clonogenic survival, indicating a mechanism of radiosensitization independent of its cytotoxic effect on cancer cells. From a clinical standpoint, enhanced radioresponse at 2 Gy is favorable as radiotherapy for primary HNSCC tumors is delivered in daily fractions of 2 Gy [38]. Our results are in line with a previous study describing a radiosensitizing effect of β-Thujaplicin on the clonogenic survival of osteosarcoma cells within similar dose ranges [19].

Induction of cell cycle arrest and apoptosis plays an integral part in mediating the anti-cancerous effect of ionizing radiation. However, cancer cells of epithelial origin are generally less sensitive to radiation-induced apoptosis compared to other types of cancer [39]. Additionally, CAL27 and FADU cell lines both harbor mutations in the TP53 gene [40]. Mutations of TP53, the gene encoding the tumor suppressor p53, are the most common genetic alteration in HNSCC and recent studies suggest mutation rates of up to 85% in HPV-negative disease [41]. p53 plays a crucial role in regulating cell cycle arrest and apoptosis and mutational dysregulation contributes to the development of radioresistant phenotypes [41, 42]. Therefore, pharmacological induction of cell cycle arrest and apoptosis may improve radiosensitivity in p53-mutated HNSCC. Using flow cytometry-based cell cycle analysis, we observed a dose dependent response in both cell lines. β-Thujaplicin led to S-phase cell cycle arrest at doses of 5 µM and 10 µM and increased the sub-G1 population at 10 µM, which is indicative of DNA fragmentation during apoptosis [31]. These results are in line with previous finding that β-Thujaplicin leads to S-phase arrest and apoptosis in other cancer cell lines [14–16]. Interestingly, β-Thujaplicin has also been shown to inhibit homologous recombination and DNA maintenance methylation, which both occur during S-phase, in dose ranges between 5 µM and 10 µM [19, 20].

Finally, we investigated the effect of β-Thujaplicin on cell migration. In advanced HNSCC, locoregional control can be achieved by means of chemoradiation and/or surgery, however, many patients eventually experience locoregional recurrence [43]. Anti-migratory effects of cancer drugs are, therefore, desirable to prevent locoregional lymphatic spread and distant metastasis. β-Thujaplicin has previously been reported to inhibit cell migration in vitro and to reduce tumor metastasis in vivo in non-HNSCC models [17, 18]. Using a wound healing assay, we were able to show that cell migration was reduced in both HNSCC cell lines after β-Thujaplicin treatment. Additionally, we demonstrated that β-Thujaplicin was effective in enhancing the anti-migratory effect of radiation on HNSCC cell lines.

In conclusion, we found that β-Thujaplicin acts as a radiosensitizer and leads to S-phase cell cycle arrest, an increased sub-G1 population indicative of apoptosis, as well as reduced migration in two established HNSCC cell lines. Based on our findings, we propose β-Thujaplicin as a promising candidate for further preclinical and clinical investigation regarding its application as radiosensitizer in HNSCC therapy.

## Data Availability

The datasets used and/or analyzed during the current study are available from the corresponding author on reasonable request.
